# Supplementary material for: Sub-Immunosuppressive Tacrolimus Ameliorates Amyloid-Beta and Tau Pathology in 3xTg-AD Mice
Source: Int J Mol Sci. 2025 Feb 20;26(5):1797. doi: 10.3390/ijms26051797 (PMC11898583; doi:10.3390/ijms26051797)

Supplementary Figure 1: Tacrolimus (TAC) concentrations measured via LC-MS/MS in whole blood (n = 4/group) from C57BL/6J mice 6-hours after receiving a single dose of TAC (1 mg/kg or 10 mg/kg) or vehicle.

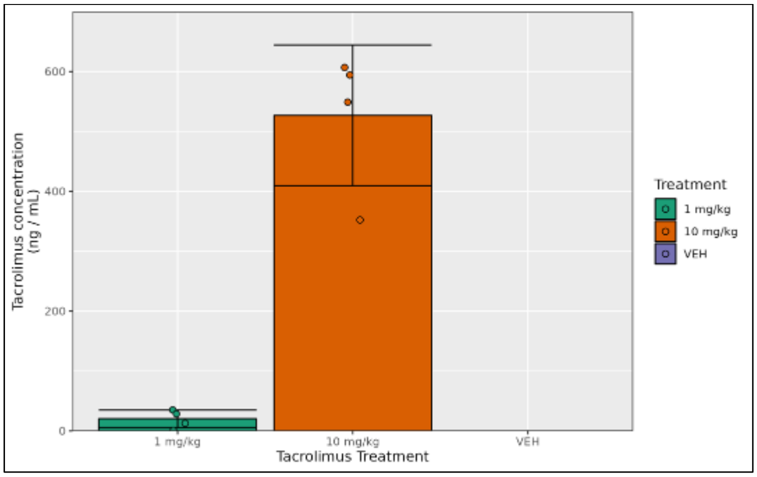

Tacrolimus Treatment	Avg. Concentration (ng / mL)
1 mg/kg	20.3 ± 14.7
10 mg/kg	527.0 ± 117.5
VEH	n.d.

n.d. = not detected or below detection limits

Supplementary Figure 2: A) Linear curve of TAC from 0.5-500 ng/mL in matrix-matched blood for calculating TAC concentrations from blood samples. Peak area ratio is calculated from the peak areas of TAC spiked into mouse blood at various concentrations with the peak areas of the internal standard [^{13}C-D_2] TAC. B) Linear curve of TAC from 0.25-25 ng/mL in matrix-matched brain extract for calculating TAC concentrations from brain samples. Peak area ratio is calculated from the peak areas of TAC spiked at various concentrations into 1.5 mg/mL brain extract background divided by the peak areas of [^{13}C-D_2] TAC. C) TAC standard at 5 ng/mL in matrix-matched brain extract to show chromatographic peak at 1.5 min. D) Example chromatogram of TAC from mouse brain sample extract.

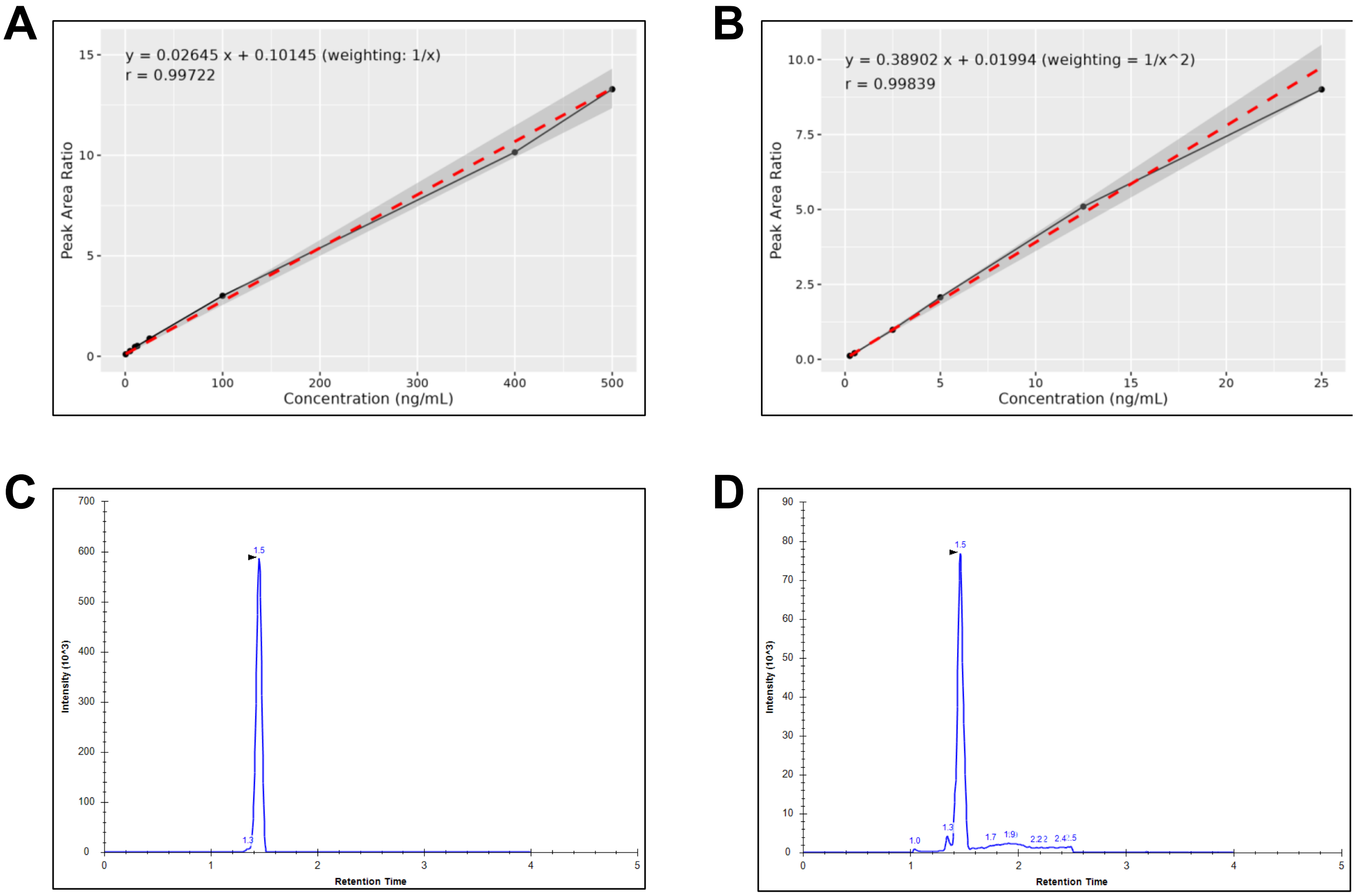

Supplementary Figure 3: Tacrolimus (TAC) administered at 0.1 mg/kg reduces amyloid-beta and tau protein levels in the hippocampus (analyzed cumulatively across CA1, CA3, and DG regions) of 3xTg-AD mice. Quantitative analyses of integrated density reveal reduced levels of amyloid-beta and phosphorylated tau in TAC-treated mice (n = 4) compared to vehicle-treated mice (n = 4).

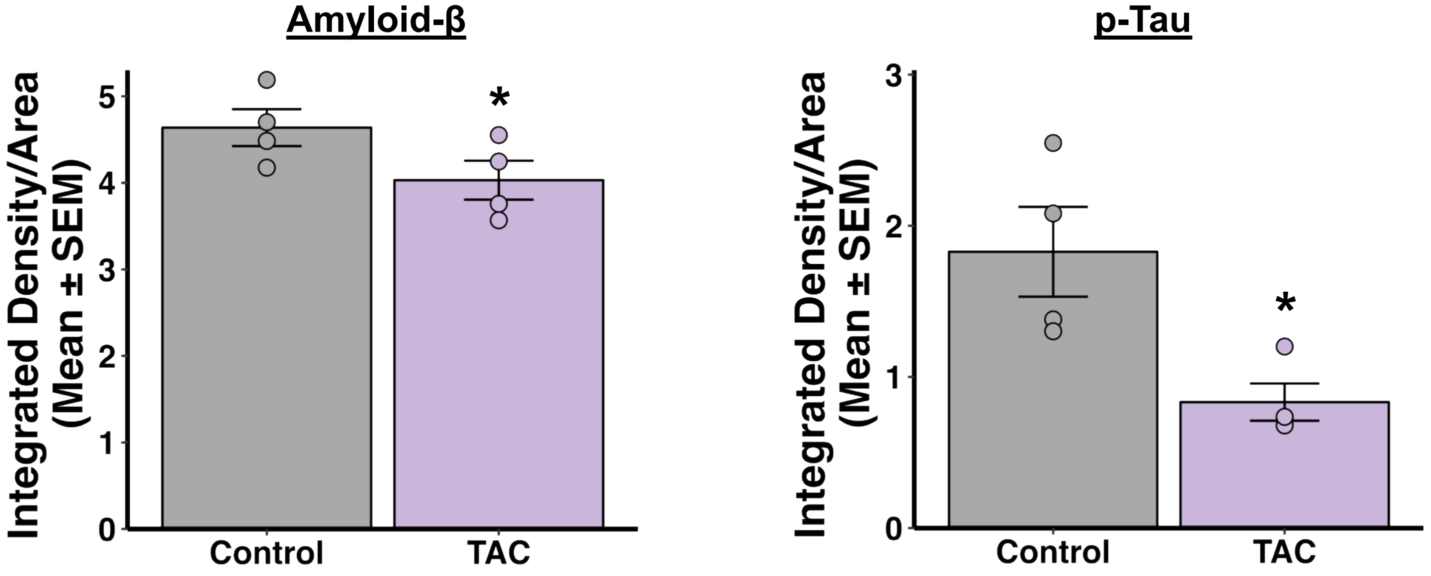

Supplement: Supplementary file 1 [file ijms-26-01797-s001.zip › ijms-3453252-supplementary.pdf]
